# Intermittent Administration of Parathyroid Hormone 1–34 Enhances Osteogenesis of Human Mesenchymal Stem Cells by Regulating Protein Kinase Cδ

**DOI:** 10.3390/ijms18102221

**Published:** 2017-10-24

**Authors:** Shu-Wen Kuo, Marilyn G. Rimando, Yi-Shiuan Liu, Oscar K. Lee

**Affiliations:** 1 Institute of Clinical Medicine, National Yang-Ming University, Taipei 11221, Taiwan; swkuo@vghtpe.gov.tw; 2 Department of Medical Research, Taipei Veterans General Hospital, Taipei 11217, Taiwan; 3 College of Science, University of Santo Tomas, Manila 1008, Philippines; mrimando7@yahoo.com; 4 Stem Cell Research Center, National Yang-Ming University, Taipei 11221, Taiwan; 5 Department of Orthopaedics and Traumatology, Taipei Veterans General Hospital, Taipei 11217, Taiwan; 6 Taipei City Hospital, Taipei 10341, Taiwan

**Keywords:** human mesenchymal stem cells, osteogenesis, parathyroid hormone, PKCδ

## Abstract

Human mesenchymal stem cells (hMSCs) can differentiate into osteoblasts and are regulated by chemical cues. The recombinant N-terminal (1–34 amino acids) fragment of the parathyroid hormone (PTH (1–34)) is identified to promote osteogenesis. The osteoanabolic effects of intermittent PTH (1–34) treatment are linked to a complex consisting of signaling pathways; additionally, protein kinase C (PKC) act as mediators of multifunctional signaling transduction pathways, but the role of PKC δ (PKCδ), a downstream target in regulating osteoblast differentiation during intermittent administration of PTH (1–34) is less studied and still remains elusive. The purpose of this study is to examine the role of PKCδ during intermittent and continuous PTH (1–34) administration using osteoblast-lineage-committed hMSCs. Relative gene expression of osteoblast-specific genes demonstrated significant upregulation of *RUNX2*, *type I Collagen*, *ALP*, and *Osterix* and increased alkaline phosphatase activity in the presence of PTH (1–34). Intermittent PTH (1–34) administration increased PKC activity at day 7 of osteogenic differentiation, whereas inhibition of PKC activity attenuated these effects. In addition, the specific isoform PKCδ was activated upon treatment. These findings demonstrate that intermittent PTH (1–34) treatment enhances the osteogenesis of hMSCs by upregulating osteoblast-specific genes via PKCδ activation.

## 1. Introduction

Bone homeostasis is achieved by a balance between bone formation and bone resorption. The rate and quality of bone formation depends on osteoblast activity, during which mineralized matrix components are secreted. In contrast, bone resorption depends on osteoclast activity to degrade tissues. An imbalance between these processes can lead to osteoporosis. The most common treatment for osteoporosis is anti-resorptive agents that inhibit osteoclast activity. Another therapeutic option, especially for severe cases, is teriparatide, a synthetic and active fragment of parathyroid hormone (PTH), comprising the N-terminal 1–34 amino acids (PTH (1–34)) [1,2]. Currently, PTH is a more effective treatment option reported to have catabolic effects on bone. The intermittent administration of this recombinant form of human PTH has been demonstrated to stimulate more bone formation than bone resorption, increasing the mineral density. However, the associated mechanisms are poorly understood [3,4].

PTH is a polypeptide hormone with 84 amino acids and is secreted by the chief cells of the parathyroid gland. It plays a major role in regulating calcium and phosphorus concentrations in the extracellular fluid and blood. Specifically, it enhances calcium absorption in the intestine by increasing activated vitamin D, and increases Ca^2+^ concentration by indirectly stimulating its osteoclast-mediated release from bones during bone resorption. PTH activates the receptor activator of nuclear factor kappa B ligand (RANKL)-osteoprotegerin-RANK system, which leads to increased osteoclast formation and activity.

Synthetic recombinant human PTH (1–34) has been shown to promote bone mineral density and has been applied to treat severe osteoporosis. However, intermittent and continuous PTH administration has disparate effects [1,2,3,4,5,6]. It is well known that intermittent daily subcutaneous PTH injection can increase osteoblast activity, whereas continuous treatment generates osteoclasts that exhibit high resorptive ability, inhibits osteoblast precursor cells, and is more active in osteoclastogenesis. At the cellular level, based on gene expression profiling, these two modes of PTH treatment were found to regulate different genes; specifically, intermittent treatment favors bone formation, whereas continuous treatment supports bone resorption [3,7].

The mechanism of PTH signaling is through the PTH receptor, a G protein-coupled receptor belonging to the family of transmembrane proteins expressed in osteoblast-lineage cells [8,9,10]. Upon PTH stimulation, the PTH receptor responds classically by activating G-protein signaling cascades such as adenylate cyclase (AC) or phospholipase C (PLC) pathways, which further activates protein kinase A (PKA) or C (PKC), respectively [11]. The AC axis is mediated by the stimulatory G protein Gαs, which activates cAMP and PKA to stimulate downstream effectors. This PKA-activated axis is linked to many PTH-induced proteins such as the activator protein-1 family, runt-related transcription factor 2 (RUNX2), and the cAMP response element binding protein. PKA activation also inhibits mitogen-activated protein kinase (MAPK) and osteoblast proliferation [12]. In the phospholipase C (PLC) axis, Gαq/11 is activated, resulting in the formation of diacylglycerol (DAG) and inositol 1,4,5-trisphosphates, which further leads to activation of PKC and release of intracellular Ca^2+^, respectively [12].

PTH stimulation, via both PKA and PKC pathways, has fundamental roles in bones. For example, activation of PKA signaling results in osteogenic differentiation and bone formation [13], whereas PKC signaling can affect cell proliferation [14]. Furthermore, the anabolic effect of PTH is attributed to the stimulation of proliferation and the differentiation of precursor cells into mature osteoblasts [12]. Parathyroid hormone-related protein also prevents apoptosis in immature pre-confluent mesenchymal cells [15]. While the majority of the PTH effect on bones is linked to PKA activation, many studies have also demonstrated an important role for activated PKC in mouse osteoblast differentiation [16]. PKC is a serine/threonine protein kinase that is stimulated by activated DAG accumulation upon phospholipase C stimulation by PTH. This protein plays important roles in regulating mammalian growth, differentiation, and apoptosis, and controls the function of other proteins including p38 MAPK, ERK1/2, and JNK. PKC affects osteoblast proliferation and participates in bone remodeling. In addition, this pathway is involved in the activity of osteoblast-related genes, as it plays a major role in FGF2 stimulation and up-regulation of *Runx2* in mouse osteoblasts [17].

The PKC family consists of fifteen isozymes in humans that are divided into three groups depending on DAG or calcium requirements for activation [18,19]. For example, the novel (n) PKCs, which include the δ, ε, η, and θ isoforms, require DAG, but not Ca^2+^, for activation. Although most isoforms are activated by the PLC pathway, the cellular role of each isoform is different. For example, PKCα signaling induces proliferation of marrow-derived osteoblastic cells in primary human osteoblasts [14]. However, in mouse osteoblasts, PKCα overexpression down-regulates early osteogenic differentiation genes and alkaline phosphatase (ALP) activity and attenuates osteogenesis in mouse preosteoblastic cells, whereas PKCα inhibition increases ERK1/2 expression and promotes bone differentiation [20]. PKCα seems to suppress osteogenic differentiation. In mouse osteoblasts, PKC β and δ/θ can promote osteogenic differentiation through BMP4 [16]. PKCδ also regulates adipogenesis and osteogenesis in mesenchymal stem cells. Increased PKCδ phosphorylation promotes osteogenesis in hMSCs [5,21] and PKCδ inhibition reduces the expression of early osteogenic differentiation genes like *ALP*, whereas inhibiting other isoforms such as PKCμ increases *ALP* and type I collagen α 1 (*COL1a1*) levels. Collectively, distinct PKC isoforms exert differential effects on both proliferation and maturation in osteoblasts.

The purpose of this study is to investigate the role of PKC signaling in both intermittent and continuous PTH treatment during the osteogenic differentiation of human MSCs, and specifically to identify the functional role of PKCδ in intermittent PTH (1–34)-enhanced osteogenesis.

## 2. Results

### 2.1. Intermittent PTH (1–34) Treatment Enhances Osteogenesis

**Figure 1:**
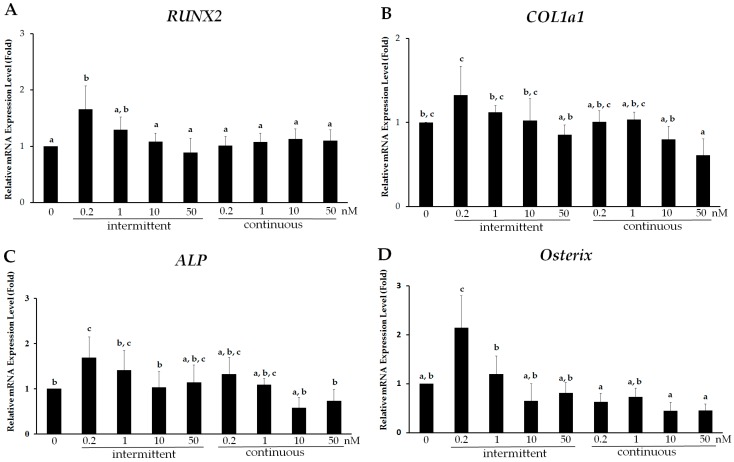
Intermittent administration of parathyroid hormone (PTH) (1–34) enhances osteogenesis in human mesenchymal stem cells (hMSCs). Relative mRNA expression of osteoblast-specific genes (**A**) *RUNX2*; (**B**) *COL1a1*; (**C**) *ALP* and (**D**) *Osterix*, assessed by real time quantitative PCR, from hMSCs on day 7 of osteogenic induction and after treatment with 0.2, 1, 10, and 50 nM PTH (1–34) using two modalities: intermittent: 2 h per day for 7 days and continuous: every 2 days for 7 days. Data are represented as mean ± SD (*n* = 4). Statistical data analysis was performed by performing one-way ANOVA with Tukey’s post-hoc tests. Different letters represent significant differences between groups; those with the same letters were not significant (*p* < 0.05).

PTH (1–34) has dual effects (anabolism or catabolism) on bone, and its osteoblastic effect depends on intermittent or continuous treatment. To investigate different concentrations and modes of PTH (1–34) treatment, hMSCs were induced to undergo osteogenic differentiation and examined on day 7 of induction. Osteogenic differentiation after PTH (1–34) treatment was assessed by measuring the expression of early osteoblast-specific genes including *RUNX2*, *COLIa1*, *ALP*, and *Osterix* by reverse transcription-quantitative polymerase chain reaction (RT-qPCR) and osteoblast activity by ALP staining. Relatively high expression of *RUNX2*, *COL1a1*, *ALP*, and *Osterix*, was observed in the intermittent PTH (1–34)-treated group compared to that in the continuous treatment group at day 7 of osteogenic differentiation (Figure 1). *RUNX2* expression at a PTH (1–34) concentration of 0.2 nM in both groups was 1.66 ± 0.42 and 1.01 ± 0.16 fold higher, respectively, than that in the control group (Figure 1A). Furthermore, *RUNX2* expression was significantly lower at higher concentrations (1, 10, and 50 nM) with no significant difference among these concentrations. Similarly, *COL1a1* expression in the intermittent group at 0.2 nM was approximately 1.33 ± 0.34 fold higher than that in the continuous group and that among different concentrations (Figure 1B). Likewise, intermittent administration of 0.2 nM PTH (1–34) resulted in 1.68 ± 0.46 and 2.14 ± 0.65 fold higher *ALP* and *Osterix* expression, respectively, compared to that with higher concentrations in the intermittent group (Figure 1C,D). In addition, cell morphology was similar at various PTH (1–34) concentrations in both intermittent and continuous groups. Based on osteoblast-specific gene expression, 2 h daily intermittent treatment of PTH (1–34) at 0.2 nM resulted in enhanced osteogenic differentiation compared to that with continuous treatment.

### 2.2. Intermittent PTH (1–34) Treatment Increases Early-Stage Osteoblast Activity during Osteogenic Differentiation

**Figure 2:**
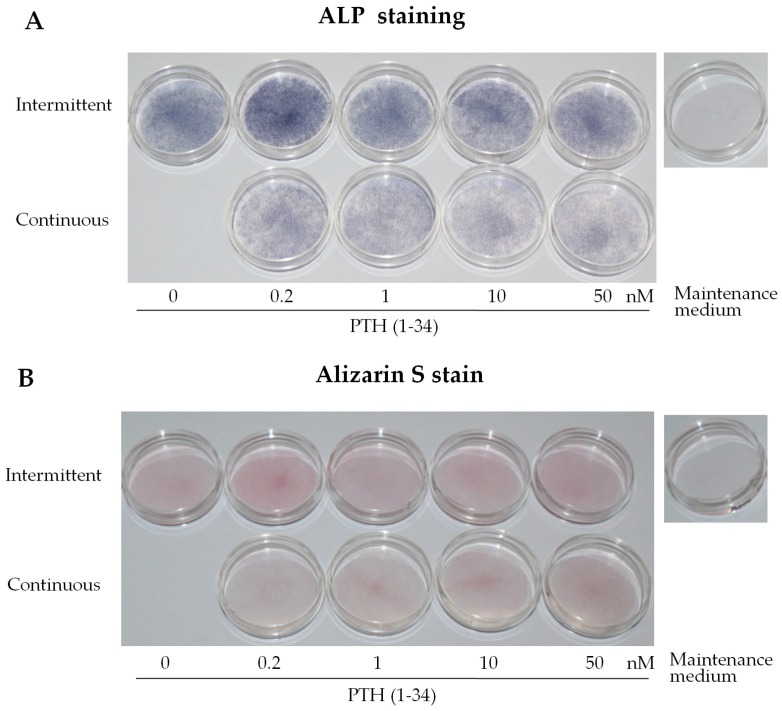

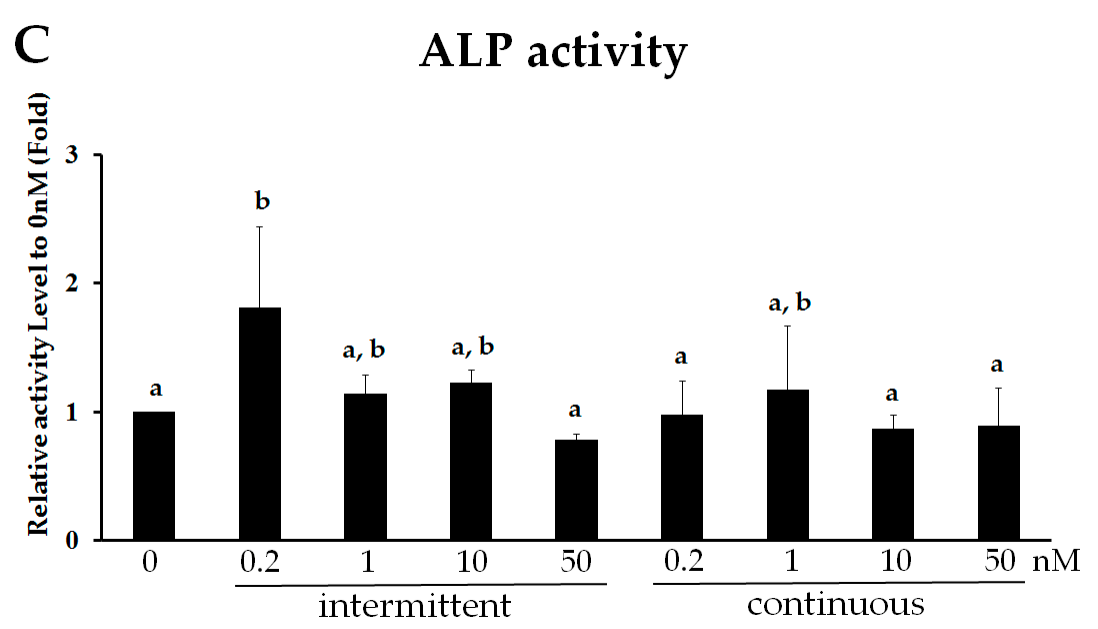
Intermittent parathyroid hormone (PTH) (1–34) treatment increased osteoblast activity during osteogenic differentiation of human mesenchymal stem cells (hMSCs). (**A**) Alkaline phosphatase (ALP) staining of hMSCs at day 7 of osteogenic induction and after treatment with 0.2, 1, 10, and 50 nM PTH (1–34) using two modalities: intermittent: 2 h per day for 7 days; and continuous: every 2 days for 7 days. Cells with ALP activity stained blue; (**B**) Alizarin Red staining for mineralized deposits in hMSCs at day 7 of osteogenic differentiation and after intermittent and continuous treatment with various concentrations (nM) of PTH (1–34); (**C**) Relative ALP activity in hMSCs at day 7 of osteogenic differentiation and after intermittent and continuous treatment with various concentrations (nM) of PTH (1–34). The relative activity in the samples was determined and compared to that of the no PTH (1–34) treatment control group and normalized to the total protein concentration (units/mg). Data are represented as mean ± SD (*n* = 3). Statistical data analysis was performed by performing one-way ANOVA with Tukey’s post-hoc tests. Different letters represent significant differences between groups; those with the same letters were not significant (*p* < 0.05).

Intermittent PTH (1–34) treatment was further confirmed to enhance osteogenesis through functional assays for ALP activity and mineralization at day 7 of osteogenic induction. To further confirm the optimal concentration, ALP activity in differentiated osteoblast precursors was examined. Enhanced activity was observed with 0.2 nM intermittent PTH (1–34) treatment compared to that in the continuous and high concentration groups (Figure 2A). Alizarin Red staining, indicating matrix mineralization (i.e., late stage differentiation), showed no significant differences between the groups (Figure 2B). These results demonstrate that intermittent PTH (1–34) at 0.2 nM significantly enhances early-stage osteoblast activity compared to that with continuous treatment. Figure 2C shows a 1.81-fold increase in ALP activity with intermittent PTH (1–34) treatment at 0.2 nM, relative to that in the control (0 nM) group. Intermittent PTH (1–34) at 0.2 nM resulted in the highest ALP activity. Thus, 0.2 nM intermittent administration, 2 h daily for 7 days, can enhance pre-osteoblast/osteoblast activity.

### 2.3. Intermittent PTH (1–34) Activates the PKC Pathway

**Figure 3:**
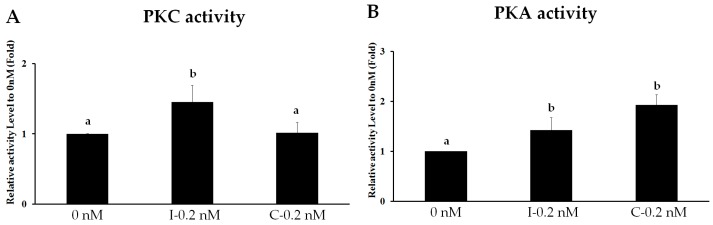
PKC pathway is activated by intermittent PTH (1–34). Protein kinase activity of PKC (**A**) and PKA (**B**) in human mesenchymal stem cells at day 7 of osteogenic differentiation after intermittent (I-0.2 nM) treatment for 2 h per day for 7 days or continuous (C-0.2 nM) treatment for 2 days for 7 days with 0.2 nM PTH (1–34). Relative kinase activity was normalized to the concentration of crude protein and compared to that in the 0-nM group. Data are represented as mean ± SD (*n* = 3). Statistical data analysis was performed by performing one-way ANOVA with Tukey’s post-hoc tests. Different letters represent significant differences between groups; those with the same letters were not significant (*p* < 0.05).

Because the PTH receptor is involved in the activation of both PKA and PKC pathways, and because PKC signaling plays a role in osteoblast differentiation [17], we assessed whether intermittent PTH (1–34) administration promotes osteogenic differentiation by activating PKC signaling. Before determining the specific involvement of PKC during osteogenic differentiation, we evaluated differential PKA and PKC activation by PTH (1–34). For this, we used the previously determined optimum concentration of 0.2 nM with intermittent and continuous administration. Intermittent PTH (1–34) treatment activated PKC activity (1.45-fold increase) compared to that in the continuous and untreated control groups (Figure 3A). In addition, 0.2 nM PTH (1–34) stimulated PKA activity in both the intermittent (1.43-fold) and continuous (1.93-fold) groups compared to that in the control group (Figure 3B). However, no significant difference in PKA activity was observed between the intermittent and continuous groups. Thus, PTH stimulated both the PKC and PKA pathway; however, the PKC-axis responded differently to intermittent PTH administration during osteogenic differentiation.

### 2.4. PKC Is Involved in Intermittent PTH (1–34)-Enhanced Osteogenesis

**Figure 4:**
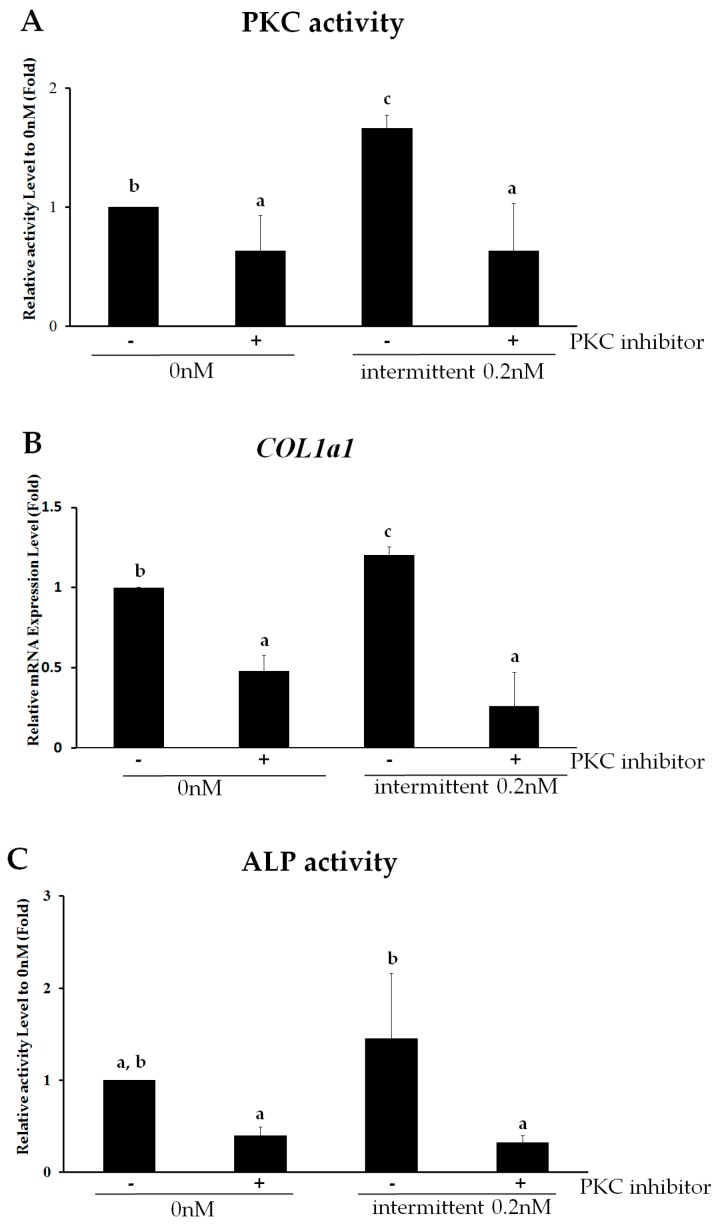
The PKC pathway is involved in intermittent parathyroid hormone (PTH) (1–34)-enhanced osteogenesis from human mesenchymal stem cells (hMSCs). (**A**) Protein kinase activity of PKC activity in hMSCs on day 7 of osteogenic differentiation after intermittent treatment with or without 0.2 nM of PTH (1–34) in the presence or absence of the broad-spectrum PKC inhibitor R136; (**B**) Relative mRNA expression of the osteoblast-specific gene *COL1a1* in hMSCs after 0.2 nM intermittent PTH (1–34) treatment in the presence or absence of R136; (**C**) Alkaline phosphatase (ALP) activity in hMSC on day 7 of osteogenic differentiation treated intermittently with or without 0.2 nM of PTH (1–34) in the presence or absence of R136. The relative activity of each sample is reported as the ratio of ALP activity to the corresponding protein concentration (units/mg). Data are represented as mean ± SD (*n* = 3). Statistical data analysis was performed by performing one-way ANOVA with Tukey’s post-hoc tests. Different letters represent significant differences between groups; those with the same letters were not significant (*p* < 0.05).

To further confirm that PKC mediates the effect of intermittent PTH (1–34) on osteogenic differentiation, signaling was blocked by the broad-spectrum PKC inhibitor R136. Relative to control levels, PKC activity decreased from 1.67-fold higher with PTH (1–34) treatment to 0.63-fold with concomitant PKC inhibition (Figure 4A), confirming that the PKC inhibitor can effectively block PKC signaling in the untreated control group. Upon PKC pathway inhibition, the expression of the osteogenic marker COL1a1 decreased (Figure 4B). Whereas intermittent PTH (1–34) treatment increased COL1a1 and ALP expression, R136 treatment decreased COL1a1 expression from 1.21-fold to 0.26-fold (relative to control levels) similar to that observed in the untreated group. ALP activity also decreased from 1.45-fold to 0.32-fold (relative to control levels) upon PKC inhibition (Figure 4C). Thus, intermittent PTH (1–34) administration promotes osteogenesis in hMSCs via PKC signaling.

### 2.5. PKCδ Mediates Intermittent PTH (1–34)-Enhanced Osteogenesis of hMSCs

**Figure 5:**
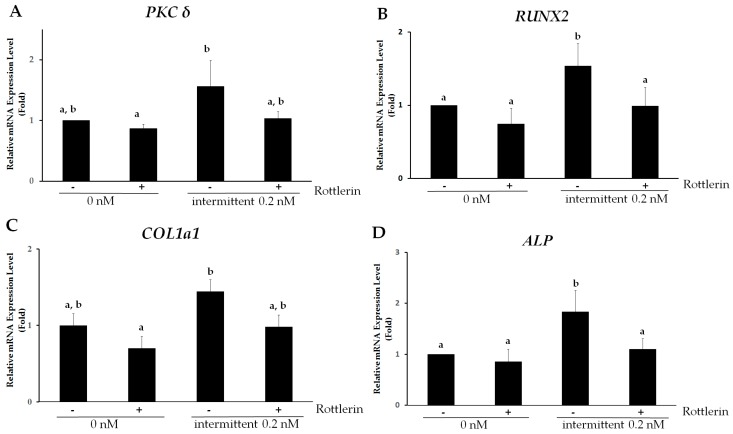

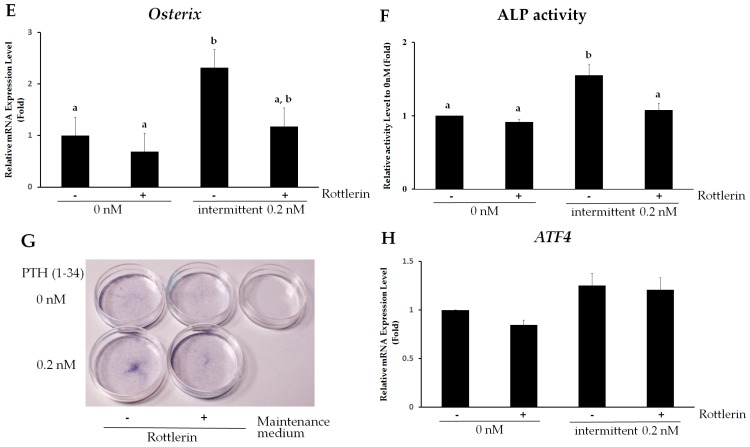
PKCδ mediates intermittent parathyroid hormone (PTH) (1–34)-enhanced osteogenesis in human mesenchymal stem cells (hMSCs). (**A**–**E**) Relative quantitative gene expression of (**A**) *PKCδ*; (**B**) *RUNX2*; (**C**) *COL1a1*; (**D**) *ALP* and (**E**) *Osterix* in hMSCs on day 7 of osteogenic differentiation after intermittent treatment with or without 0.2 nM of PTH (1–34) in the presence or absence of the specific PKCδ inhibitor Rottlerin. Data are presented as mean ± SD (*n* = 3). Different letters represent significant differences between groups (*p* < 0.05). (**F**,**G**) Functional assay for osteogenic differentiation after intermittent treatment with or without 0.2 nM of PTH (1–34) in the presence or absence of Rottlerin using hMSCs on day 7 of osteogenic induction. (**F**) Relative alkaline phosphatase (ALP) activity of the sample is reported as the ratio of ALP activity to the corresponding protein concentration (units/mg). Data are represented as mean ± SD (*n* = 3). Statistical data analysis was performed by performing one-way ANOVA with Tukey’s post-hoc tests. Different letters represent significant differences between groups; those with the same letters were not significant (*p* < 0.05); (**G**) ALP staining in hMSCs on day 7 of osteogenic induction and after intermittent treatment with 0.2 nM PTH (1–34) and Rottlerin; (**H**) Relative quantitative gene expression of *ATF4* in hMSCs on day 7 of osteogenic differentiation after intermittent treatment with or without 0.2 nM of PTH (1–34) in the presence or absence of the specific PKCδ inhibitor Rottlerin.

Since PKC signaling is involved in enhanced osteogenic differentiation mediated by intermittent PTH (1–34) treatment, we examined the role of specific PKC isoforms in osteogenesis. PKCα and PKCδ have been reported to be involved in osteogenesis from hMSCs [5,20,21]. We initially examined *PKCδ* and *PKCα* expression in differentiating hMSCs. Figure 5A shows that *PKCδ* was expressed in differentiating hMSCs; expression increased by 1.56-fold upon intermittent administration of 0.2 nM PTH (1–34). However, PKCα expression did not increase with treatment. To determine whether PKCδ specifically mediates enhanced osteogenesis with intermittent PTH (1–34) treatment, hMSCs were treated with the specific PKCδ inhibitor Rottlerin on day 7 of osteogenic induction. Expression of *PKCδ* declined to 1.03-fold (relative to control levels) upon treatment with Rottlerin (Figure 5A).

To further confirm that the PKCδ pathway directly regulates the expression of osteogenic genes upon intermittent PTH (1–34) treatment, gene expression of early osteoblast markers was examined in the presence of Rottlerin. Osteogenic-specific gene expression of *RUNX2*, *COL1a1*, *ALP*, and *Osterix*, originally 1.65-, 1.44-, 1.84-, and 2.32-fold higher than that of the control, respectively, decreased to 1.00-, 0.98-, 1.10-, and 1.18-fold, compared to expression in the control group in hMSCs on day 7 of osteogenic differentiation with 0.2 nM of PTH (1–34) treatment (Figure 5B–E). In the same conditions, ALP activity decreased with Rottlerin treatment (1.55 ± 0.14-fold in the intermittent PTH group vs. 1.08 ± 0.10-fold with Rottlerin, compared to expression in the untreated group) (Figure 5F). Similarly, decreased ALP activity was observed in the presence of a specific PKCδ inhibitor (Figure 5G). These results demonstrate the specific involvement of PKCδ in intermittent PTH (1–34)-induced early stage osteogenesis.

We further examined whether activating transcription factor 4 (ATF4) is involved in PKCδ-mediated promotion of osteogenic differentiation upon intermittent PTH treatment. ATF4 is a leucine zipper-containing transcription factor that plays a critical role in the anabolic action of PTH [22]. ATF4 also regulates the expression of late osteogenic marker osteocalcin [22,23]. Although gene expression of *ATF4* in hMSCs slightly increased after 0.2 nM PTH (1–34) treatment compared to the control group at day 7 of osteogenic differentiation , ATF4 expression was unaffected upon PKCδ inhibition (Figure 5H). Thus, ATF4 may not be involved in the PKCδ action in enhancing osteogenesis during intermittent PTH treatment. The result indicated that the action of intermittent PTH (1–34)-enhanced osteogenesis of hMSCs mediated by PKCδ is mainly targeted in the early stage of osteogenesis.

## 3. Discussion

PTH (1–34) is one of the clinically approved treatment regimens for severe osteoporosis due to its ability to stimulate bone formation. It is well known that PTH exerts variable effects depending on the mode of PTH administration. For example, it can stimulate bone formation and increase bone density when administered intermittently, but it results in the opposite catabolic effect if administered continuously [24]. Therefore, we aimed to determine the concentration and mode of PTH (1–34) exposure that can optimally promote osteogenesis. The experimental strategy was to compare the two modes of PTH (1–34) treatment, and to determine the dose, based on the range reported in the literature [25,26,27]. The concentration used herein (0.2–50 nM) falls within the range previously reported [14,27,28]. Since the normal physiological blood concentration of full length PTH (PTH 1–84) is 10–60 pg/mL (approximately 1.1–6.3 pM) and the clinical dose of PTH (1–34) is 20 µg (60 µM) per day for humans [2], both the physiological and clinical doses were used as the basis for determining the appropriate concentration in this study. In addition, the intermittent treatment time was designed considering that based on pharmacokinetics, PTH (1–34) has an absorption time of less than or within 60 min with a half-life of 1 h [29]. In addition, PTH (1–34) injection will metabolically decline to endogenous baseline levels within hours, similarly to that reported by others for PTH (1–34) [25,27,30]. Therefore, in this study, intermittent treatment was selected with a duration of exposure for 2 h.

Our results showed that intermittent low-dose (0.2 nM) PTH (1–34) enhanced osteogenesis, compared to that with continuous and high dose treatment (Figure 1). High concentrations of PTH (1–34) did not efficiently promote hMSC osteogenic differentiation compared to that with low doses. This suggests that PTH (1–34) does not have a dose-dependent effect on osteogenic differentiation, and that higher PTH concentrations might even reduce bone differentiation. In addition, intermittent was shown to be far better than continuous administration. These results are in agreement with reports of others [30,31]. However, these studies used different PTH (1–34) doses; thus, the selection of dose might vary depending on source and stem cell type.

A range of PTH administration modalities differentially affect osteoblasts. Intermittent PTH administration is associated with increased osteoblastic activity [32]; however, continuous treatment affects both bone resorptive osteoclasts and osteoblast precursor cells by activating and inhibiting their activities, respectively. Our results showed that human MSCs respond favorably to PTH (1–34) at low and intermittent doses, which promotes the formation of differentiating osteoblast precursor cells. Our findings concur with other studies using different cell types that clearly demonstrated the relationship between PTH and osteogenesis. In the osteoblast precursor cell line C3H10T1/2, for example, PTH promotes osteoblast differentiation; in addition, animal studies have confirmed the effect of PTH on osteoblasts in bone tissues, thus establishing a role for PTH in bone formation [33]. In addition, daily subcutaneous injection of PTH (1–34) for three weeks in rats resulted in a higher proportion of osteoprogenitor cells, which expressed higher levels of the osteoblast-specific marker ALP [34].

Although intermittent PTH increases bone formation in humans, the mechanism is still not fully understood [3,7]. The use of different PTH fragments has resulted in different cellular responses, and this has provided insights into the signaling pathways linked to PTH activation. For example, synthetic fragments of PTH can activate PKC and PKA pathways through G protein Gαq (PLC) and Gαs (AC) with different potencies. Shorter fragments of PTH (1–34) or substitution of some residues increases affinity, potency, and selectivity by modulating the AC pathway [35]. Specifically, daily injection of human PTH (1–34), but not PTH (1–31), stimulated both PKA and PKC activity in osteoblast-like ROS 17/2 rat osteosarcoma cells [25], which was found to account for the anabolic action of PTH [36]. Furthermore, the active N-terminus of PTH (3–34) was shown to activate the PKA pathway, but not PKC, whereas both pathways were inactivated by the 30–34 N-terminus of PTH [37]. In our study, we specifically found that PKC axis activation is dependent on the mode of administration (intermittent) of PTH (1–34), and this pathway was implicated in promoting osteogenesis from hMSCs.

Although our results demonstrated that PTH (1–34) stimulates both PKC and PKA pathways, both responded differently depending on the mode of treatment. Intermittent administration resulted in enhanced PKC activity compared to that with continuous treatment, whereas no statistically significant difference was observed regarding PKA signaling between intermittent and continuous modes. This might be attributed to the transient activation of PKC after exposure to PTH (1–34). This agent can quickly induce PKC activity, but activity is subsequently reduced to baseline levels, whereas increased PKA activity is sustained during prolonged PTH exposure. This might also depend on the fragment size of PTH, since high affinity binding between PTH and its receptor can elicit different signaling responses; for example, PTH (1–34)-mediated signaling is shorter, with a decay time of approximately 2 h [38]. PTH absorption and elimination might also account for this effect. Thus, PTH receptor signaling might be sustained for different durations, thereby triggering different signaling responses. In addition, studies have shown that different PTH analogues and PTH receptors produce different conformational changes, and are induced by different G proteins and PKA or PKC pathways [39].

In Figure 3, intermittent PTH (1–34) treatment tended to increase PKC activity to a greater extent than continuous administration. Although continuous treatment also slightly increased PKC activity, PKA activity tended to increase with continuous treatment compared to that with intermittent treatment. Studies have shown that different concentrations of PTH (1–34) will differentially stimulate PKC and PKA activity; for example, low concentrations stimulate PKC translocation, but high concentrations strongly stimulate and favor PKA activity [40]. Prolonged PTH (1–34) exposure, for different durations, results in different degrees of PKC pathway stimulation [41]. Some studies have reported that PTH (1–34) exposure for 24 or 48 h results in the activation of the PKC pathway [42]. Therefore, PTH (1–34) activation, resulting in stimulation of either the PKC or PKA pathway, might be dependent on and influenced by PTH (1–34) treatment time, dose, absorption, and elimination or half-life. Our results have shown that intermittent 0.2 nM PTH (1–34) administration selectively activates the PKC pathway, more so than PKA.

In addition, PTH regulation can activate multiple signal transduction pathways and PTH is a biphasic regulator of PKA and PKC signaling [43]. Furthermore, at the cellular level, based on gene expression profiling, these two modes of PTH treatment were found to regulate different genes; specifically, intermittent treatment favors bone formation, whereas continuous treatment results in bone resorption [3,7]. Our study suggests that the acute responses to PTH (1–34) elicit a transient and stronger stimulation of the PKC pathway, which promotes osteogenesis of MSCs. Although we cannot rule out the existence of a role for the PKA pathway and dependence on PTH (1–34) treatment time for osteogenesis, the PKC pathway is most likely involved in intermittent administration of PTH. It is thus likely that different PTH treatment modalities can elicit different degree and duration of response on both PKA and PKC signaling.

PKC is a multifunctional cell signal transduction pathway and can regulate many signaling responses to modulate functions such as proliferation, differentiation, apoptosis, and survival [44]. Our results and others confirm that the PKC axis is involved in osteogenesis, as it was shown to modulate *RUNX2* [45] and *ALP* expression [16]. However, there are fifteen different PKC isoforms, and it is not clear whether each isoform plays similar roles in bone metabolism. Among the different isoforms, PKCδ and PKCα were reported to be involved in osteogenesis in hMSCs [12,15,19]. However, it was suggested that these isoforms have opposing effects. For example, increased PKCδ activity inhibited expression of PKCα during osteogenic induction of hMSCs [21]. In addition, PKCδ was shown to induce *Runx2* expression, promote osteogenic differentiation in mouse osteoblastic cells [17], and induce expression of *ALP* and *COL1a1* [5]; in addition, PKCδ homozygous mutant mice show reduced Osterix activity in the early embryonic skeleton [46]. Our study therefore focused on these two isoforms, and of these it was found that PKCδ responds to intermittent PTH (1–34) treatment during osteogenesis. Whether PTH regulates *cis*- or *trans*-acting elements specific for PKCδ alone, and not on PKCα, requires further exploration. Furthermore, we found that intermittent PTH treatment through PKCδ enhances the early stage of osteogenesis but not the later stages, as seen in the mineralization assay, with no difference in the expression of late markers osteopontin, osteocalcin. ATF4 is known to play critical roles in the anabolic effect of PTH and regulate osteocalcin expression [22,23]. Although a slightly increased *ATF4* expression was observed, it was not inhibited by the PKCδ inhibitor, suggesting that the enhancement of osteogenesis by PTH is more pronounced in the early stage and there is no difference in the later stages. Nonetheless, it has been shown that the amino acid length, mode of administration, and concentration are significant factors in influencing the pathway and mechanistic response of MSCs to PTH.

Taken together, the osteogenic differentiation of hMSCs is enhanced by PTH (1–34) at low and intermittent doses, and this involves enhanced activation of the PKC axis, and not PKA signaling, by specifically regulating PKCδ. This study presents an important role for PKCδ in regulating bone differentiation and provides insights into the associated mechanism. This may affect the selection of PTH delivery mode and fragment size for future clinical applications.

## 4. Materials and Methods

### 4.1. Cell Culture and Osteogenic Differentiation of hMSCs

Human adipose-derived mesenchymal stem cells (hMSCs) were purchased from Steminent Biotherapeutics Inc. (Taipei, Taiwan). hMSCs were maintained in expansion medium (MesenPRO RS™, Invitrogen, Grand Island, NY, USA) supplemented with 100 units/mL of penicillin, 1000 units/mL of streptomycin, and 2 mmol/L l-glutamine (Sigma-Aldrich, St. Louis, MO, USA), and incubated at 37 °C, 5% CO_2_ and 95% relative humidity. To induce osteogenic differentiation, hMSCs were cultured with osteogenic induction medium (OIM) consisting of Iscove’s Modified Dulbecco’s medium (Gibco, Grand Island, NY, USA) supplemented with 10 mM β-glycerol phosphate (Sigma-Aldrich, St. Louis, MO, USA), 0.1 µM dexamethasone (Sigma-Aldrich, St. Louis, MO, USA), and 0.2 mM ascorbic acid (Sigma-Aldrich, St. Louis, MO, USA), and the medium was changed every three days.

### 4.2. PTH (1–34) Treatment

The recombinant form of Human Parathyroid hormone N-terminus 1–34 amino acid; PTH (1–34) was purchased as Teriparatide (Forteo^®^) (Lilly, Indianapolis, IN, USA). PTH (1–34) treatment on hMSCs were divided into two groups: Intermittent (I) and Continuous (C). For intermittent treatment, hMSCs under OIM were treated with PTH (1–34) for two hours every day for seven days. After 2 h of treatments, the cells were washed and replaced with fresh OIM. For continuous treatment, hMSCs were treated with PTH (1–34) for two days in OIM for seven days. PTH (1–34) titration was initially performed using various concentrations (0.2, 1, 10, and 50 nM) to determine the optimum concentration to be used for subsequent assays. Phosphate-buffered saline (PBS) was used as vehicle-control group.

### 4.3. Inhibitors

A broad-type PKC inhibitor (R136, Ro 31-822, Sigma-Aldrich, St. Louis, MO, USA) was dissolved in H_2_O to a final working concentration of 5 μm. Rottlerin (a specific inhibitor of PKCδ, Cat#R5648, Sigma-Aldrich, St. Louis, MO, USA) was dissolved in DMSO to a final working concentration of 2 μm.

### 4.4. Reverse Transcription and Quantitative Real-Time Polymerase Chain Reaction (RT-qPCR)

Gene expression of osteoblast-specific makers during different stages of osteoblast maturation was examined. The expression levels of these genes upon treatment with various concentrations PTH (1–34) were assessed by RT-qPCR. A total of 3 × 10^5^ hMSC cells were treated with various PTH (1–34) concentration for 7 days. Total RNA was extracted using TRIzol reagent (Invitrogen) and 2 µg of RNA samples were reversed transcribed using MMLV Reverse Transcriptase 1st-Strand cDNA synthesis Kit (EPICENTRE^®^ Biotechnologies, Madison, WI, USA). Subsequent PCR amplification was performed by Quantitative real-time PCR using Step OnePlus™ Real-Time PCR System platform (ABI, Thermo Fisher Scientific, Waltham, MA, USA). Intron spanning primers specific to each gene were designed with corresponding probes from the Universal ProbeLibrary Assay Design Center (Roche Applied Science, Mannheim, Germany) and were detected using Universal ProbeLbrary (Roche) (Table 1). The average threshold cycle (*C*_t_) value for each gene was normalized by that of glyceraldehyde 3-phosphate dehydrogenase (GAPDH).

### 4.5. Alkaline Phosphatase (ALP) Staining

hMSCs were treated with PTH(1–34) for 7 days, washed with PBS, and fixed with 3.7% paraformaldehyde (Sigma-Aldrich) at room temperature for five minutes. Alkaline phosphatase (ALP) staining was performed using NBCT/BCIP (5-bromo-4-chloro-3-indolyl phosphate/4-nitroblue tetrazolium) (Sigma-Aldrich, B1911) according to manufacturer’s instruction. Briefly, the fixed cells were stained with NBCT/BCIP at room temperature for one hour and stained cells were imaged by digital camera (Alpha 350, Sony, Tokyo, Japan). The blue-stained cells demonstrate ALP expression and the absence of ALP expression will result in no stain.

### 4.6. Alkaline Phosphatase (ALP) Activity Assay

ALP activity was quantified using Alkaline Phosphatase Assay Kit (Fluorimetric) (ab83371, Abcam, Cambridge, UK) according to the manufacturer’s instruction. Briefly, hMSCS were homogenized in assay buffer and centrifuged to remove insoluble material at 13,000× *g*; then, the lysate was collected. 100 µL of the sample was added into 96-well plates, followed by incubation with 20 µL of 0.5 mM MUP substrate for 30 min at 25 °C and protection from light. A 20 µL of stop solution was added into each well, and fluorescence intensity was measured at EX/EM 360/440 using a fluorescence microtiter plate reader (SpectraMax M5, Molecular Devices, Sunnyvale, CA, USA). A standard curve was generated by ALP enzyme that convert MUP to fluorescent 4-Methylumbelliferone (4-MU). The standard curve readings were plotted and applied to the sample readings to get the amount of 4-MU generated from the PTH (1–34)-treated samples. The ALP activity was determined by *A*/*V*/*T* (mU/mL), where *A* is the amount of 4-MU generated by sample (in nmol), *V* is volume of sample added in the assay well (in mL), and *T* is reaction time (in min). The total protein concentrations were determined using protein assay reagent (Bio-Rad, Hercules, CA, USA). The relative activity of the sample is reported as the ratio of activity and the corresponding protein concentration (unit/mg).

### 4.7. Protein Kinase C (PKC) Kinase Activity Assay

The PKC activity was quantified using PKC kinase activity assay kit (ADI-EKS-420A, Enzo Life Sciences, Farmingdale, NY, USA). The PKC kinase assay is specific for all isoforms PKC (PKCα, β, γ, δ, ε, μ, θ, ζ) and detects PKC phosphotransferase activity by ATP. PKC activity was detected in hMSCs under intermittent treatment with PTH (1–34) and with inhibitors for PTH (1–34). Protein lysates were collected from hMSCs and added with protease and kinase inhibitors. A 0.2–2 μg concentration of total protein were added into each of the wells. The wells were pre-coated with PKC substrate which gets phosphorylated by the active PKC. Subsequently, ATP was added into each well to initiate the reaction, followed by the addition of phosphospecific substrate antibody and conjugate HRP antibody. The activity assay was developed with tetramethylbenzidine substrate (TMB) and the relative change in PKC phosphotransferase activity was measured at an absorbance of OD (450 nm). The relative kinase activity was computed by subtracting the hMSCs lysate sample absorbance minus blank absorbance and was normalize to the concentration of the crude protein per assay.

### 4.8. Protein Kinase A (PKA) Kinase Activity Assay

PKA activity was determined and quantified using PKA Kinase Activity Assay Kit (ADI-EKS-390A, Enzo Life Sciences, NY, USA) according to the manufacturer’s instructions. The kinase activity was developed with tetramethylbenzidine substrate (TMB) substrate and color development stopped by acid, and read at 450 nm with the color intensity proportional to PKA phosphotransferase activity.

### 4.9. Statistical Analysis

Statistical analysis was performed by one-way ANOVA and Tukey’s post hoc test using the PSPP statistical software (Version 0.10.4, http://www.gnu.org/software/pspp/; GNU Operating System). The data were presented as mean ± standard deviation (SD) from at least two independent experiments. A *p*-value of <0.05 was considered statistically significant. All experiments were repeated more than three times.

## 5. Conclusions

The recombinant N-terminal 1–34 amino acid fragment of the PTH influences osteoblast activity in differentiating human MSCs. However, the concentration, mode of treatment, and duration of exposure can influence the overall PTH response of differentiating osteoprogenitors. Intermittent treatment at a low dose of 0.2 nM enhanced osteogenesis and favored osteoblast activity, compared to those with high and continuous treatment. Furthermore, this acute response to PTH (1–34) elicited transient and stronger stimulation of the PKC pathway, more so than the PKA axis. Although both PKC and PKA pathways were stimulated by PTH (1–34) and were involved in osteoblast differentiation, PKC was found to be involved, enhancing osteogenesis during intermittent PTH treatment. Furthermore, PKCδ was identified to mediate this PTH (1–34)-induced enhanced osteogenesis. This study provides insights into how modulating the dose and treatment duration activates different signaling cascades and also, how intermittent PTH treatment can positively modulate bone formation by enhancing osteoblast-specific gene expression and activity by activating the PKC axis and, specifically, how PKCδ is elucidated. Such a finding may contribute to the future development of anti-osteoporotic anabolic agents.

## Figures and Tables

**Table 1:** Primer sequences and probes from the Universal Probe Library used for RT-qPCR.

Gene Name	Oligonucleotide Sequence	Probe Number
*RUNX2*	5′–CTACCACCCCGCTGTCTTC–3′/5′–CAGAGGTGGCAGTGTCATCA–3′	29
*COL1a1*	5′–ATGTTCAGCTTTGTGGACCTC–3′/5′–CTGTACGCAGGTGATTGGTG–3′	15
*ALP*	5′–AGAACCCCAAAGGCTTCTTC–3′/5′–CTTGGCTTTTCCTTCATGGT–3′	31
*Osterix*	5′–GACTGCAGAGCAGGTTCCTC-3′/5′–TAACCTGATGGGGTCATGGT–3′	43
*PKCδ*	5′–TCGACTGGGAAAAACTGGAG–3′/5′–CTTGGTTGGTTCCCTTTCCA–3′	80
*PKCα*	5’–ATTATCCCCGCTGGATCAC–3′/5′–CTCTGCTCCTTTGCCACAC–3′	83
*ATF4*	5′–TGGTCAGTCCCTCCAACAAC–3′/5′–CTATACCCAACAGGGCATCC–3′	88
*GAPDH*	5′–GCTCTCTGCTCCTCCTGTTC–3′/5′–ACGACCAAATCCGTTGACTC–3′	60

## Abbreviations

MSCs	Mesenchymal stromal cells
PTH	Parathyroid hormone
PKC	Protein kinase C
PKCδ	Protein kinase C δ
PKCα	Protein kinase C α
RUNX2	Runt-related transcription factor 2
COL1a1	Collagen type 1 a1
ALP	Alkaline phosphatase
OIM	Osteogenic induction medium
ATF4	Activating transcription factor 4

## References

[1] Osagie-Clouard L., Sanghani A., Coathup M., Briggs T., Bostrom M., Blunn G. (2017). Parathyroid hormone 1–34 and skeletal anabolic action: The use of parathyroid hormone in bone formation. Bone Jt. Res..

[2] Neer R.M., Arnaud C.D., Zanchetta J.R., Prince R., Gaich G.A., Reginster J.Y., Hodsman A.B., Eriksen E.F., Ish-Shalom S., Genant H.K. (2001). Effect of parathyroid hormone (1–34) on fractures and bone mineral density in postmenopausal women with osteoporosis. N. Engl. J. Med..

[3] Silva B.C., Bilezikian J.P. (2015). Parathyroid hormone: Anabolic and catabolic actions on the skeleton. Curr. Opin. Pharmacol..

[4] Greenhill C. (2017). Parathyroid function: Action of parathyroid hormone in osteocytes. Nat. Rev. Endocrinol..

[5] Liu Q., Wan Q., Yang R., Zhou H., Li Z. (2012). Effects of intermittent versus continuous parathyroid hormone administration on condylar chondrocyte proliferation and differentiation. Biochem. Biophys. Res. Commun..

[6] Aslan D., Andersen M.D., Gede L.B., de Franca T.K., Jorgensen S.R., Schwarz P., Jorgensen N.R. (2012). Mechanisms for the bone anabolic effect of parathyroid hormone treatment in humans. Scand. J. Clin. Lab. Investig..

[7] Silva B.C., Costa A.G., Cusano N.E., Kousteni S., Bilezikian J.P. (2011). Catabolic and anabolic actions of parathyroid hormone on the skeleton. J. Endocrinol. Investig..

[8] Juppner H. (1994). Molecular cloning and characterization of a parathyroid hormone/parathyroid hormone-related peptide receptor: A member of an ancient family of G protein-coupled receptors. Curr. Opin. Nephrol. Hypertens..

[9] Vilardaga J.P., Romero G., Friedman P.A., Gardella T.J. (2011). Molecular basis of parathyroid hormone receptor signaling and trafficking: A family B GPCR paradigm. Cell. Mol. Life Sci..

[10] Cheloha R.W., Gellman S.H., Vilardaga J.P., Gardella T.J. (2015). PTH receptor-1 signalling-mechanistic insights and therapeutic prospects. Nat. Rev. Endocrinol..

[11] Datta N.S., Abou-Samra A.B. (2009). PTH and PTHrP signaling in osteoblasts. Cell. Signal..

[12] Swarthout J.T., D’Alonzo R.C., Selvamurugan N., Partridge N.C. (2002). Parathyroid hormone-dependent signaling pathways regulating genes in bone cells. Gene.

[13] Siddappa R., Martens A., Doorn J., Leusink A., Olivo C., Licht R., van Rijn L., Gaspar C., Fodde R., Janssen F. (2008). cAMP/PKA pathway activation in human mesenchymal stem cells in vitro results in robust bone formation in vivo. Proc. Natl. Acad. Sci. USA.

[14] Lampasso J.D., Marzec N., Margarone J., Dziak R. (2002). Role of protein kinase C α in primary human osteoblast proliferation. J. Bone Miner. Res..

[15] Chen H.L., Demiralp B., Schneider A., Koh A.J., Silve C., Wang C.Y., McCauley L.K. (2002). Parathyroid hormone and parathyroid hormone-related protein exert both pro- and anti-apoptotic effects in mesenchymal cells. J. Biol. Chem..

[16] Park K.H., Han D.I., Rhee Y.H., Jeong S.J., Kim S.H., Park Y.G. (2010). Protein kinase C βII and δ/θ play critical roles in bone morphogenic protein-4-stimulated osteoblastic differentiation of MC3T3-E1 cells. Biochem. Biophys. Res. Commun..

[17] Kim H.J., Kim J.H., Bae S.C., Choi J.Y., Kim H.J., Ryoo H.M. (2003). The protein kinase C pathway plays a central role in the fibroblast growth factor-stimulated expression and transactivation activity of Runx2. J. Biol. Chem..

[18] Mellor H., Parker P.J. (1998). The extended protein kinase C superfamily. Biochem. J..

[19] Newton A.C. (2003). Regulation of the ABC kinases by phosphorylation: Protein kinase C as a paradigm. Biochem. J..

[20] Nakura A., Higuchi C., Yoshida K., Yoshikawa H. (2011). PKCα suppresses osteoblastic differentiation. Bone.

[21] Lee S., Cho H.Y., Bui H.T., Kang D. (2014). The osteogenic or adipogenic lineage commitment of human mesenchymal stem cells is determined by protein kinase C δ. BMC Cell Biol..

[22] Yu S., Franceschi R.T., Luo M., Fan J., Jiang D., Cao H., Kwon T.G., Lai Y., Zhang J., Patrene K. (2009). Critical role of activating transcription factor 4 in the anabolic actions of parathyroid hormone in bone. PLoS ONE.

[23] Yang X., Karsenty G. (2004). ATF4, the osteoblast accumulation of which is determined post-translationally, can induce osteoblast-specific gene expression in non-osteoblastic cells. J. Biol. Chem..

[24] Uzawa T., Hori M., Ejiri S., Ozawa H. (1995). Comparison of the effects of intermittent and continuous administration of human parathyroid hormone(1–34) on rat bone. Bone.

[25] Ogita M., Rached M.T., Dworakowski E., Bilezikian J.P., Kousteni S. (2008). Differentiation and proliferation of periosteal osteoblast progenitors are differentially regulated by estrogens and intermittent parathyroid hormone administration. Endocrinology.

[26] Di Bernardo G., Galderisi U., Fiorito C., Squillaro T., Cito L., Cipollaro M., Giordano A., Napoli C. (2010). Dual role of parathyroid hormone in endothelial progenitor cells and marrow stromal mesenchymal stem cells. J. Cell. Phys..

[27] Tian Y., Xu Y., Fu Q., He M. (2011). Parathyroid hormone regulates osteoblast differentiation in a Wnt/β-catenin-dependent manner. Mol. Cell. Biochem..

[28] Sammons J., Ahmed N., El-Sheemy M., Hassan H.T. (2004). The role of BMP-6, IL-6, and BMP-4 in mesenchymal stem cell-dependent bone development: Effects on osteoblastic differentiation induced by parathyroid hormone and vitamin D_3_. Stem Cells Dev..

[29] Satterwhite J., Heathman M., Miller P.D., Marin F., Glass E.V., Dobnig H. (2010). Pharmacokinetics of teriparatide (rhPTH[1–34]) and calcium pharmacodynamics in postmenopausal women with osteoporosis. Calcif. Tissue Int..

[30] Rickard D.J., Wang F.L., Rodriguez-Rojas A.M., Wu Z., Trice W.J., Hoffman S.J., Votta B., Stroup G.B., Kumar S., Nuttall M.E. (2006). Intermittent treatment with parathyroid hormone (PTH) as well as a non-peptide small molecule agonist of the PTH1 receptor inhibits adipocyte differentiation in human bone marrow stromal cells. Bone.

[31] Zhou S., Bueno E.M., Kim S.W., Amato I., Shen L., Hahne J., Bleiberg I., Morley P., Glowacki J. (2011). Effects of age on parathyroid hormone signaling in human marrow stromal cells. Aging Cell.

[32] Rubin M.R., Cosman F., Lindsay R., Bilezikian J.P. (2002). The anabolic effects of parathyroid hormone. Osteoporos. Int..

[33] Dobnig H. (2004). A review of teriparatide and its clinical efficacy in the treatment of osteoporosis. Expert Opin. Pharmacother..

[34] Nishida S., Yamaguchi A., Tanizawa T., Endo N., Mashiba T., Uchiyama Y., Suda T., Yoshiki S., Takahashi H.E. (1994). Increased bone formation by intermittent parathyroid hormone administration is due to the stimulation of proliferation and differentiation of osteoprogenitor cells in bone marrow. Bone.

[35] Shimizu M., Potts J.T., Gardella T.J. (2000). Minimization of parathyroid hormone. Novel amino-terminal parathyroid hormone fragments with enhanced potency in activating the type-1 parathyroid hormone receptor. J. Biol. Chem..

[36] Hu Y., Chan E., Wang S.X., Li B. (2003). Activation of p38 mitogen-activated protein kinase is required for osteoblast differentiation. Endocrinology.

[37] Fujimori A., Cheng S.L., Avioli L.V., Civitelli R. (1992). Structure-function relationship of parathyroid hormone: Activation of phospholipase-C, protein kinase-A and -C in osteosarcoma cells. Endocrinology.

[38] Dean T., Vilardaga J.P., Potts J.T., Gardella T.J. (2008). Altered selectivity of parathyroid hormone (PTH) and PTH-related protein (PTHrP) for distinct conformations of the PTH/PTHrP receptor. Mol. Endocrinol..

[39] Gesty-Palmer D., Chen M., Reiter E., Ahn S., Nelson C.D., Wang S., Eckhardt A.E., Cowan C.L., Spurney R.F., Luttrell L.M. (2006). Distinct β-arrestin- and G protein-dependent pathways for parathyroid hormone receptor-stimulated ERK1/2 activation. J. Biol. Chem..

[40] Janulis M., Tembe V., Favus M.J. (1992). Role of protein kinase C in parathyroid hormone stimulation of renal 1,25-dihydroxyvitamin D3 secretion. J. Clin. Investig..

[41] Iida-Klein A., Varlotta V., Hahn T.J. (1989). Protein kinase C activity in UMR-106–01 cells: Effects of parathyroid hormone and insulin. J. Bone Miner. Res..

[42] Guimaraes G.N., Rodrigues T.L., de Souza A.P., Line S.R., Marques M.R. (2014). Parathyroid hormone (1–34) modulates odontoblast proliferation and apoptosis via PKA and PKC-dependent pathways. Calcif. Tissue Int..

[43] Halladay D.L., Miles R.R., Thirunavukkarasu K., Chandrasekhar S., Martin T.J., Onyia J.E. (2001). Identification of signal transduction pathways and promoter sequences that mediate parathyroid hormone 1–38 inhibition of osteoprotegerin gene expression. J. Cell. Biochem..

[44] Mochly-Rosen D., Das K., Grimes K.V. (2012). Protein kinase C, an elusive therapeutic target?. Nat. Rev. Drug Discov..

[45] Yang D., Singh R., Divieti P., Guo J., Bouxsein M.L., Bringhurst F.R. (2007). Contributions of parathyroid hormone (PTH)/PTH-related peptide receptor signaling pathways to the anabolic effect of PTH on bone. Bone.

[46] Tu X., Joeng K.S., Nakayama K.I., Nakayama K., Rajagopal J., Carroll T.J., McMahon A.P., Long F. (2007). Noncanonical Wnt signaling through G protein-linked PKCδ activation promotes bone formation. Dev. Cell.

